# Utilization of dual catalysts for high-yield boron nitride nanotube synthesis via chemical vapor deposition

**DOI:** 10.55730/1300-0527.3772

**Published:** 2025-11-05

**Authors:** Şaban KALAY

**Affiliations:** Division of Biochemistry, Department of Basic Pharmaceutical Sciences, Faculty of Pharmacy, University of Health Sciences, İstanbul, Turkiye

**Keywords:** Boron nitride nanotube, chemical vapor deposition, template, dual catalyst, colemanite, B_2_O_3_

## Abstract

The direct synthesis of boron nitride nanotubes (BNNTs) via chemical vapor deposition (CVD) in high-temperature furnaces remains highly challenging due to difficulties in optimizing key experimental parameters such as synthesis temperature and catalyst composition. These challenges often result in uncontrolled growth behavior, adversely affecting the quality and yield of the BNNTs. In this study, colemanite was effectively utilized as a boron source for the high-yield synthesis of directionally aligned BNNTs. The synthesis was carried out using a CVD method that used a dual-catalyst system comprising Fe_2_O_3_ and MgO in conjunction with a silicon carbide template under high-temperature conditions. The resulting BNNTs were characterized using scanning electron microscopy and high-resolution transmission electron microscopy, as well as spectroscopic methods including Fourier-transform infrared spectroscopy, Raman spectroscopy, X-ray diffraction, and X-ray photoelectron spectroscopy. This innovative CVD strategy offers a cost effective and efficient way to produce high-purity BNNTs from colemanite, significantly expanding their potential for various applications.

## 1. Introduction

Boron nitride nanotubes (BNNTs) are structural analogues of carbon nanotubes (CNTs), with the key distinction being that BNNTs are composed of boron and nitrogen atoms instead of carbon. The partially ionic character of the B–N bond imparts BNNTs with greater resistance to oxidation and high-temperature environments, surpassing that of CNTs [1–5]. Furthermore, BNNTs can absorb neutrons [6], broadening their application potential in aerospace technologies such as satellites and rocket systems [7,8]. BNNTs have a wide band gap that, unlike CNTs, remains unaffected by variations in chirality, diameter, or wall number [9]. Their exceptional elastic modulus and mechanical flexibility have also facilitated their incorporation into advanced composite materials [10–15]. BNNTs can interact through π–π stacking with polymers containing aromatic rings, enabling their dispersion in organic solvents like chloroform tetrahydrofuran and thus allowing for the development of BNNT-polymer composites [11]. Another notable characteristic of BNNTs is their hydrogen storage capacity. Recent studies report that BNNTs can adsorb nearly twice as much hydrogen as CNTs, with adsorption levels reaching up to 0.85% [16]. Additionally, BNNTs hold promise for applications in microfluidic devices at the nanoscale [17].

The diverse biological applications of BNNTs highlight their promising potential for future biomedical use as well. BNNTs with piezoelectric properties have been used in bone tissue engineering studies [18] while hydroxyapatite BNNT scaffolds promote osteogenic activity [19]. Polyethyleneimine (PEI)-coated BNNTs were tested on human neuroblastoma cell line and acceptable cell viability was observed at concentrations up to 5.0 μg/mL of PEI-BNNTs in the culture medium [20]. Due to their ability to load drugs at levels exceeding 300 times their own weight, BNNTs are also considered suitable carriers for chemotherapeutic agents [21]. Furthermore, investigations have shown that BNNTs do not induce oxidative DNA damage or apoptosis in planarian stem cells, indicating their biocompatibility and potential use in de novo tissue regeneration without eliciting adverse effects [22].

BNNTs can be used in sensor technologies, neutron capture therapy, biomaterial applications, drug delivery, biomolecule immobilization, defense, and space industries. BNNTs incorporated with gold nanoparticles have shown remarkable humidity detection properties expressed as 100 s/15 s [23]. The addition of 2% BNNTs into a polyimide composite resulted in approximately a 460% increase in electroactive behavior and sensing efficiency, indicating their potential as highly responsive sensing materials [24].

Beyond sensing applications, BNNTs possess significant neutron capture capacity that has been explored for the therapeutic management of cerebral glioblastoma multiforme [25]. A β-1,3-glucan and HER-2-antibody conjugated BNNT complex achieved nearly 30-fold higher neutron capture therapy compared with clinical agents [26].

For biomedical applications, BNNTs are typically encapsulated or coated with biocompatible polymers to enhance stability and cellular interactions. Polycaprolactone-coated BNNTs have been incorporated into orthopedic implants and had positive effects on cell growth and viability [27]. BNNTs have also been integrated into bone tissue scaffolds to provide structural reinforcement [28,29]. Incorporating 4% BNNTs into hydroxyapatite composites improved elasticity by 120%, hardness by 129%, and fracture toughness by 86% [28]. Furthermore, BNNTs had favorable interactions with bone cells and maintained an acceptable level of biocompatibility [29]. These findings collectively highlight their promise as reinforcing components for orthopedic, skeletal, and next generation biocomposite systems. Covalent conjugation of BNNTs with the copolymer poly(acrylic acid-co-fluorescein acrylate) (P(AA-co-FA)) resulted in the formation of pH-sensitive, fluorescent, water-dispersible hybrid nanomaterials [30]. The functionalized BNNTs had strong green fluorescence and were efficiently internalized by human normal prostate epithelial cells (PNT1A) and prostate cancer cells (DU145), indicating their potential applicability in cellular imaging studies [30].

In pharmaceutical contexts, BNNTs have functioned as effective nanocarriers for intracellular delivery and controlled release applications [31]. Both the inner cavity and outer surface of BNNTs were successfully utilized for curcumin loading, ensuring a sustained and well-regulated release profile [31]. Additionally, BNNTs have been reported to be a highly effective immobilization matrix. Pristine BNNTs could bind bioactive macromolecules such as ferritin, cytochrome c, glucose oxidase, and streptavidin [32]. Studies conducted under supersonic hot jet environments showed that BNNT-reinforced hybrid composites had outstanding resistance to wear and ablation [33]. Compared with conventional materials, these composites had superior thermal protection and mechanical stability [33]. BNNTs retained their structural integrity under extreme pressure and temperature, confirming their feasibility for use in rocket exhaust assemblies and spacecraft thermal shielding layers [33]. Moreover, BNNT-based composites had strong electromagnetic radiation shielding capabilities [34]. Their combination of low density and high mechanical strength enables the fabrication of lightweight but durable composite architectures. Experimental evidence further indicated that layered BNNT structures optimize radiation absorption while enhancing mechanical durability. Taken together, these observations establish BNNT-reinforced nanocomposites as next-generation multifunctional materials suitable for both structural reinforcement and radiation protection in high performance aerospace and defense applications [33,34].

Despite their wide range of potential uses, the production of BNNTs faces significant obstacles, primarily due to the high costs associated with precursor chemicals, the requirement for challenging optimal synthesis conditions, the need for advanced synthesis equipment such as thermal plasma furnaces [35–37], and the complex purification processes required to remove residual impurities [38–39].

Colemanite (2CaO·3B_2_O_3_·5H_2_O), a boron-rich mineral that crystallizes in the monoclinic system, is among the most abundant boron source, containing about 51% B_2_O_3_. It has a Mohs hardness of 4.5 and a specific gravity of 2.42 g/cm^3^ and it is commonly used as a precursor in boric acid synthesis [40]. Due to its high boron content, economic value, and availability, colemanite and other boron minerals have recently gained attention as suitable candidates for BNNT synthesis [18,41,42]. Kalay et al. [41] were the first to report the direct synthesis of BNNTs from colemanite using Fe_2_O_3_ as a catalyst. These BNNTs were characterized as multiwalled structures with a wall spacing of 0.34 nm. Furthermore, BNNT composites synthesized from colemanite have shown enhanced piezoelectric properties and incorporated morphologies such as nanoflakes, triangular boron nitride forms, and fiber-like structures [18]. In another study, BNNTs were synthesized from colemanite using a thiol-based catalyst at 1050 °C [42].

The first synthesis of BNNTs was achieved via arc-discharge methods [43]. Subsequently, techniques such as laser ablation, ball milling, substitution reactions, and chemical vapor deposition (CVD) were explored for BNNT synthesis [44–47]. Among these, CVD is frequently used due to its scalability and high efficiency [41–43]. Ball milling can be used independently or integrated with CVD to promote homogeneous mixing of reactants through mechanical grinding. In one approach, BNNTs were synthesized using ammonia borane as the precursor in a hydrogen-assisted induction thermal plasma furnace, resulting in lower hexagonal boron nitride (h-BN) impurity content [48,49]. Bae et al. [49] used a high-temperature thermal plasma system to produce double-walled BNNTs with yields up to 60% and lengths ranging from 0.979 μm to 10.366 μm.

To reduce synthesis temperature, Wang et al. [50] used a catalyst system comprising MgO, K_2_CO_3_, and B in a molar ratio of 1:1:4 and elemental boron. Using a custom-designed reactor, they achieved BNNT formation at 850 °C via CVD. In another study, a surface-like growth strategy was developed to produce horizontally aligned BNNTs (HABNNTs), using W_2_B_5_/Zn as the precursor and a Si/SiO_2_ template [51].

In high-temperature reactors, 3 principal forces govern BNNT formation: the fluid drag force (Fda), the radial directional force induced by thermal gradients (Frd), and the thermophoretic force (Ftr) resulting from temperature differences [51,52]. By positioning Si/SiO_2_ templates at specific angles, researchers were able to reduce the random orientation of BNNTs [51]. Growth morphology is influenced by NH_3_ gas flow rate (e.g., 200 sccm), the total volume of NH_3_ gas, and the orientation of the template.

In traditional synthesis setups, substrate and catalyst mixtures are placed inside an alumina crucible. At the crucible base, all 3 forces Fda, Frd, and Ftr coexist, causing BNNTs formed in agglomerated clusters to localize beneath a plate placed over the crucible [51,53]. Researchers positioned Si/SiO_2_ templates at 60°, 90°, and 120° angles to study orientation effects using imaging and spectroscopic techniques [51]. They indicated that BNNT orientations differed after BNNT syntheses performed with Si/SiO_2_ templates placed at different angles in the alumina reaction vessel. The results indicated that angle-dependent alignment behavior, with 90° placements eliminating all but the radial force (Frd) and proving to be the most effective for HABNNT growth [51]. These findings highlight that, in addition to reducing h-BN impurities, dual-catalyst systems and a precise understanding of directional forces are key to achieving monodisperse BNNT structures.

This study was an extension of previous work and proposed, for the first time, a dual-catalyst system comprising Fe_2_O_3_ and MgO for BNNT synthesis from colemanite. This replaces costlier precursors such as ammonia borane, W_2_B_5_/Zn, and elemental boron with colemanite, offering a more economical and widely available substrate. To enhance yield, we introduced a newly designed reaction chamber model that strategically leverages the Fda, Frd, and Ftr forces. Additionally, B_2_O_3_ was used to further direct BNNT alignment. This allowed for a novel reduction of the reaction temperature to 1100 °C.

## 2. Materials and methods

### 2.1. Chemicals and reagents

Colemanite (Ca_2_B_6_O_11_·5H_2_O) and B_2_O_3_ were generously provided by Eti Mine (Ankara, Türkiye). Iron(III) oxide and magnesium oxide were purchased from Merck (Darmstadt, Germany). High-purity ammonia gas (99.98%) was sourced from Schick GmbH & Co. KG (Vaihingen an der Enz, Germany).

### 2.2. Boron nitride nanotube synthesis

Substrate–catalyst systems studied included colemanite/Fe_2_O_3_ (2 g:160 mg), colemanite/Fe_2_O_3_/MgO (2 g:160 mg:40 mg) and B_2_O_3_/Fe_2_O_3_/MgO (2 g:40 mg:40 mg). In each setup, 14 stainless steel balls were used in a ball mill (Fritsch Premium Line Pulverisette, Idar-Oberstein, Germany) at 150 rpm overnight. The next day, 8–9 mL of 27% aqueous NH_3_ was added and milling was continued for 50 min. A 500 μL aliquot of each catalyst–substrate mixture was spread as a thin film over a 4.5 × 2.5 cm silicon carbide (SiC) surface and dried with heat under NH_3_ flow in a fume hood. The thermal treatment was carried out in a high-temperature furnace (Protherm, PTF 14/50/450, Schömberg, Germany) programmed for 146 min at 1250 °C, followed by 60 min at the same temperature. Specific reactor and catalyst parameters are detailed with the corresponding results. All syntheses were conducted under a constant flow of 300 sccm high-purity ammonia gas. The BNNT synthesis experiments, using either a single- or dual-catalyst system, were repeated at least 20 times (n = 20).

### 2.3. Scanning electron microscopy analysis

Samples were mounted on carbon discs and coated with a 10 nm gold layer in an argon atmosphere using a Baltec SDC 005 sputter coater (Pfäffikon, Switzerland). Scanning electron microscopy (SEM) images were acquired using a Carl Zeiss (Oberkochen, Germany) EVO-40 system under high vacuum and accelerating voltage (10 kV), with Iprobe values adjusted between 50 and 100.

### 2.4. Transmission electron microscopy

Transmission electron microscopy (TEM) observations were performed on a JEOL-2100 (Tokyo, Japan) high-resolution transmission electron microscope (HRTEM) equipped with an Oxford Instruments (Abingdon, UK) 6498 EDS system. Analyses were conducted at an accelerating voltage of 120 kV using a LaB_6_ filament.

### 2.5. Fourier-transform infrared and Raman spectroscopy

Fourier-transform infrared (FT-IR) spectra were collected using a Thermo Fisher Scientific (Waltham, MA, USA) NICOLET IS50 system and UV–Vis measurements were carried out with a PerkinElmer (Waltham, MA, USA) Lambda 25 spectrophotometer. Raman spectra were obtained using a Renishaw (Gloucestershire, UK) InVia Reflex Raman microscope equipped with a 514 nm Ar laser (30 mW). Exposure time was set to 10 s using a 50× objective. The system was calibrated before each scan using an internal silicon standard, with spectra centered at 520 cm^−1^.

### 2.6. X-ray diffraction

X-ray diffraction (XRD) patterns were recorded with a Shimadzu (Kyoto, Japan) XRD-6000 instrument using ICDD PDF-4 software. Scans were conducted in continuous mode over a 2θ range of 2.000–69.980°, with a scan speed of 2.0000°/min, a sampling pitch of 0.0200°, and a preset time of 0.60 s.

### 2.7. X-ray photoelectron spectroscopy

X-ray photoelectron spectroscopy (XPS) was utilized to analyze the surface chemical states, using a Thermo Fisher Scientific K-α X-ray photoelectron spectrometer with an Al Kα monochromator source (1486.6 eV). Data fitting was performed using Avantage 5.9 software, with all spectra calibrated relative to the C 1s peak at 284.5 eV.

## 3. Results and discussion

**Figure 1:**
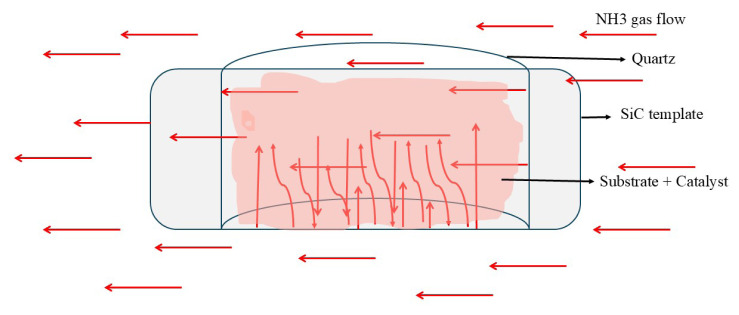
The scheme of the reaction chamber utilized during synthesis experiments. It depicts the direction of gas flow, the quartz glass plate, the SiC template, and the reaction mixture (substrate + catalyst) applied to the SiC surface.

**Figure 2:**
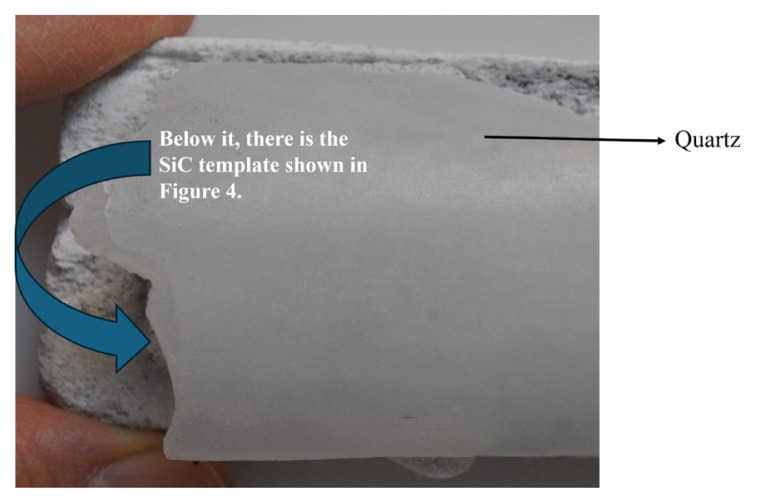
A top view of the reaction chamber showing that the quartz glass is designed to allow NH_3_ flow from multiple directions (front, back, and sides). This configuration was positioned inside a high-temperature furnace, enabling the BNNT synthesis via the CVD method.

A comprehensive understanding of the alignment mechanisms, i.e. the forces guiding BNNT growth, is essential for successful synthesis from colemanite, B_2_O_3_, and other boron minerals. To direct BNNT formation via Ftr and Fda forces, an inclined quartz glass surface was placed atop a SiC substrate (Figures 1 and 2). This setup not only facilitated BNNT deposition and collection but also prevented their displacement by thermal convection (Figure 2).

As shown in Figure 2, a quartz glass piece was positioned on top of the SiC template. NH_3_ gas can enter the system through the gaps on the side and in front of the quartz glass. If the gas fails to infiltrate adequately, BNNT synthesis cannot be achieved effectively. A portion of the NH_3_ flow also passes directly over the substrate surface, ensuring homogeneous distribution of the gas across the colemanite + catalyst system.

Due to the dominant role of Ftr and Fda growth mechanisms in BNNT formation, all synthesis operations used the chamber configuration schematized in Figure 1 and topologically represented in Figure 2. The syntheses were conducted using colemanite and B_2_O_3_ as substrates, combined with Fe_2_O_3_ and MgO catalysts, under a continuous 300 sccm flow of NH_3_ gas in a high-temperature furnace.

**Figure 3:**
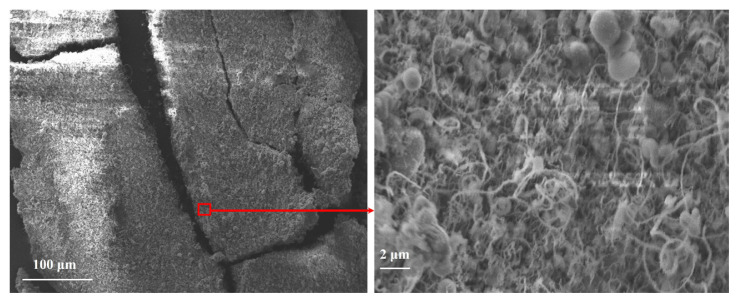
Wide (left) and magnified (right) SEM imaging of BNNTs synthesized using the colemanite and Fe_2_O_3_ system.

Figure 3 shows BNNTs synthesized from a colemanite + Fe_2_O_3_ system. These results confirm that BNNTs can be produced at 1280 °C under NH_3_ flow using the CVD technique with Fe_2_O_3_ as a catalyst. However, alongside BNNTs, the formation of h-BN crystals was also observed (Figure 3). Such h-BN impurities have been reported in previous studies and can be removed using alternative washing procedures or by using different substrate materials, although this adds to the overall cost [50,54,55].

**Figure 4:**
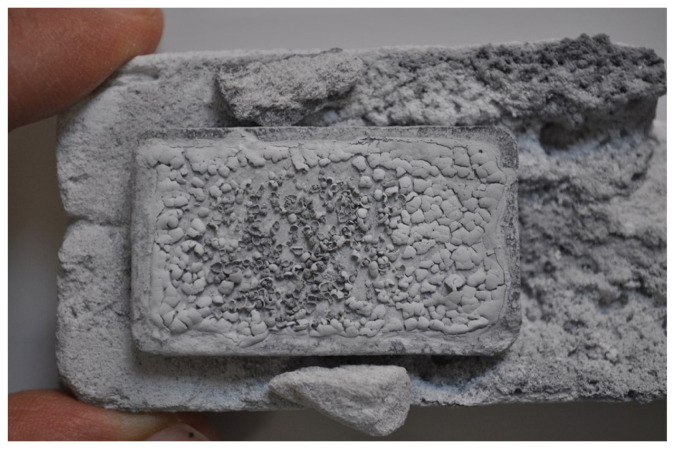
A photo of the appearance of BNNTs on the SiC template retrieved from the high-temperature furnace after CVD synthesis.

**Figure 5:**
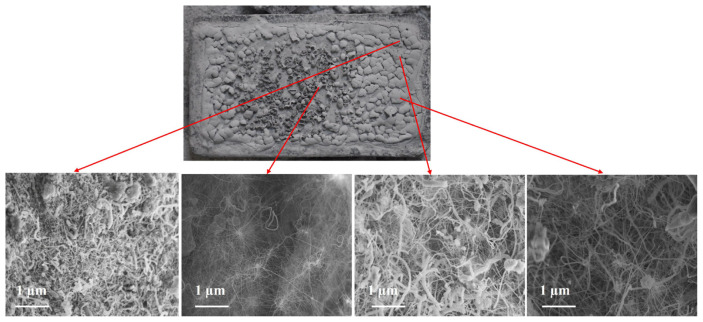
Photo and SEM images of BNNTs synthesized using the colemanite + Fe_2_O_3_ + MgO catalyst system. The upper image shows pure BNNTs on the SiC template, collected from the regions indicated by tweezers and imaged using SEM.

**Figure 6:**
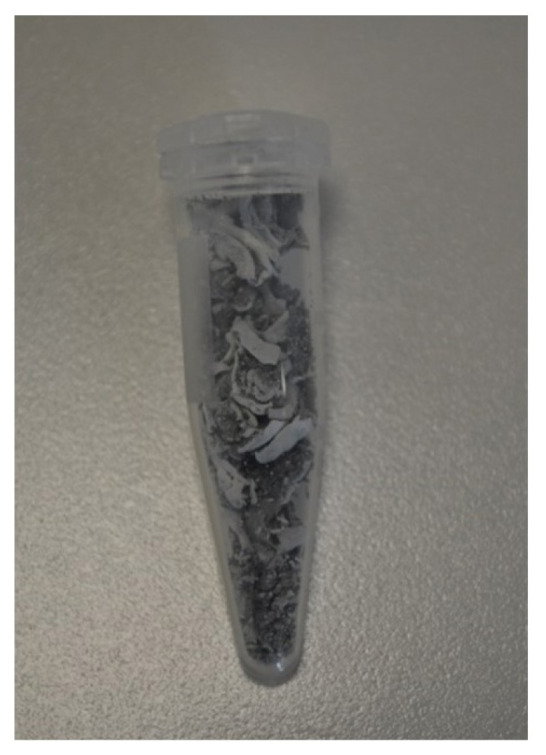
A photo of the subgram-scale isolation of pure BNNTs using Fe_2_O_3_ + MgO binary catalyst and a specially designed reaction chamber.

Following this, BNNTs were synthesized using a binary catalyst system consisting of colemanite + Fe_2_O_3_ + MgO (Figures 4 and 5). SEM showed that the presence of h-BN impurities was significantly reduced in the Fe_2_O_3_ + MgO dual-catalyst system (Figure 5). After CVD synthesis, the white product regions were selectively collected using tweezers (Figure 6). This approach enabled the cost-effective, single-step production of subgram-scale, high-purity BNNTs from colemanite.

In other studies, researchers typically mixed the substrate + catalyst mixture before thermal processing [1,30]. However, our findings indicate that applying ball milling for at least 6 h instead of dispersion significantly enhances BNNT yield. To investigate directional BNNT growth within the reaction chamber, B_2_O_3_ was used as the substrate.

**Figure 7:**
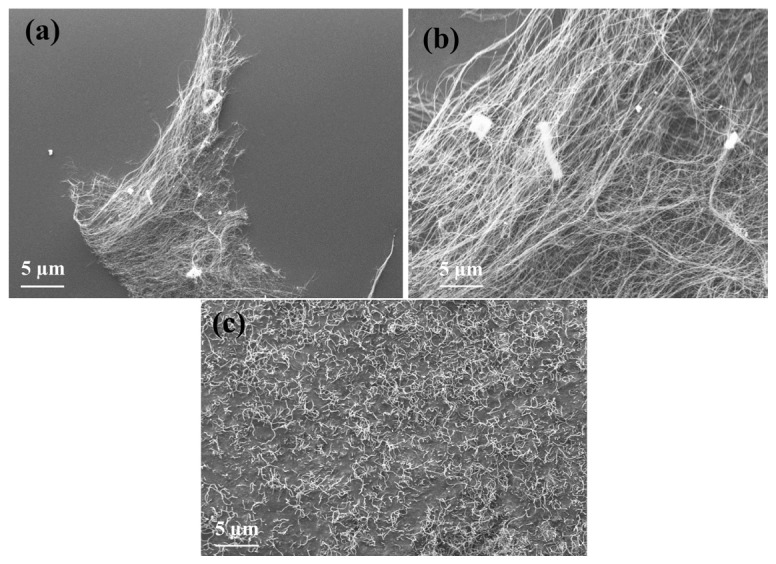
The SEM images of the BNNTs synthesized via CVD from a B_2_O_3_/Fe_2_O_3_/MgO catalyst system. High-aspect-ratio BNNTs (a, b) formed at 1250 °C for 1 h, randomly oriented BNNTs (c) formed at 1100 °C for 5 h.

BNNT synthesis was carried out using a B_2_O_3_/Fe_2_O_3_/MgO ratio of 2 g:40 mg:40 mg. The thickness of the substrate + catalyst layer applied to the SiC plate plays a crucial role in achieving high-purity BNNTs; excessive thickness increases the likelihood of impurities. After ball milling, the substrate–catalyst mixture was applied as a thin film onto a SiC plate. The mixture was then subjected to CVD at 1250 °C for 1 h under NH_3_ flow, yielding high-aspect-ratio BNNTs (Figures 7a and 7b).

In contrast, conducting the same reaction at 1100 °C for 5 h resulted in BNNTs that were shorter and randomly oriented (Figure 7c).

Using B_2_O_3_ as a substrate resulted in a high BNNT yield, with negligible formation of h-BN. Uniformly structured BNNTs were obtained. However, the overall BNNT amount was lower than that achieved using colemanite.

**Figure 8:**
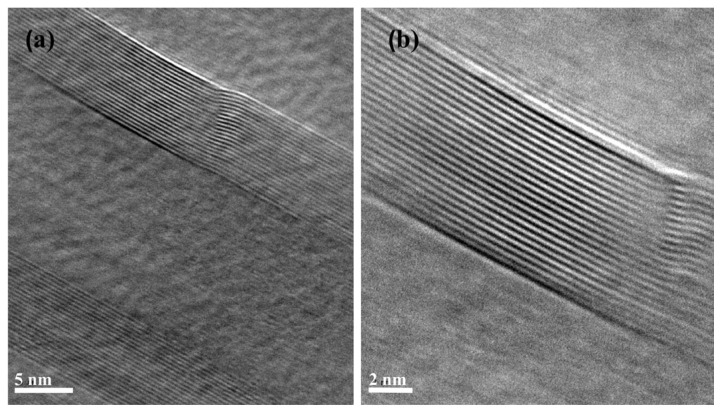
TEM and HRTEM characterization of BNNTs synthesized from colemanite. The average number of BNNT walls was measured as 22. The distance between walls was measured as 0.34 nm.

TEM of BNNTs synthesized from colemanite showed hollow nanotubes with an average diameter of approximately 27.3 nm (Figure 8). HRTEM images showed well-defined crystalline tube walls with a lattice spacing of 0.34 nm, consistent with the (002) crystal plane of h-BN. Diameter and layer number distributions confirmed an average diameter of 27.3 nm and an average wall count of 22. These findings confirm the successful synthesis of BNNTs.

**Figure 9:**
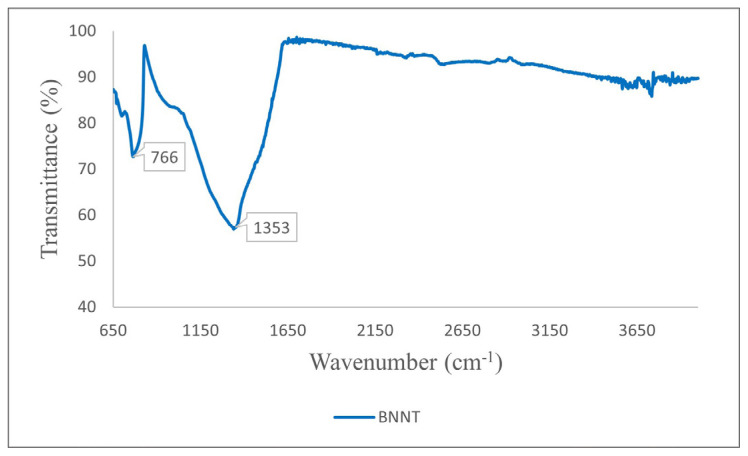
FT-IR spectra of BNNTs synthesized from colemanite. The B–N–B bending mode is observed at 766 cm^−1^, and the B–N bond vibration is observed at 1353 cm^−1^.

FT-IR spectroscopy of the synthesized BNNTs showed 2 distinct absorption bands at 766 cm^−1^ and 1353 cm^−1^ (Figure 9). The weaker band at 766 cm^−1^ corresponds to the B–N–B bending mode parallel to the c-axis, while the stronger band at 1353 cm^−1^ corresponds to B–N stretching vibrations perpendicular to the c-axis. These are characteristic peaks for h-BN [56–59]. The peak at 1353 cm^−1^ also represents the E_2_g phonon mode associated with interlayer vibrations in h-BN [53,57]. The full width at half maximum of the 1353 cm^−1^ band was 18.3 cm^−1^. This agrees with the values reported in the literature and supports the high crystallinity of the synthesized BNNTs [51].

**Figure 10:**
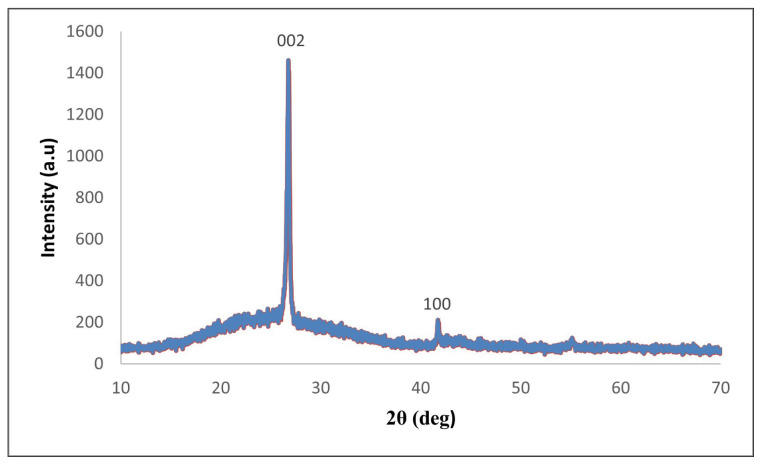
XRD analysis confirming the crystalline structure of BNNTs synthesized from colemanite. The peaks corresponding to the (002) and (100) planes indicate the BN lattice and confirm the absence of any impurities.

The purity and composition of the BNNTs were also confirmed through X-ray diffraction (XRD) analysis. The diffraction patterns had characteristic peaks corresponding to the (002) and (100) planes of h-BN, indicating the absence of undesired phases or impurities (Figure 10).

**Figure 11:**
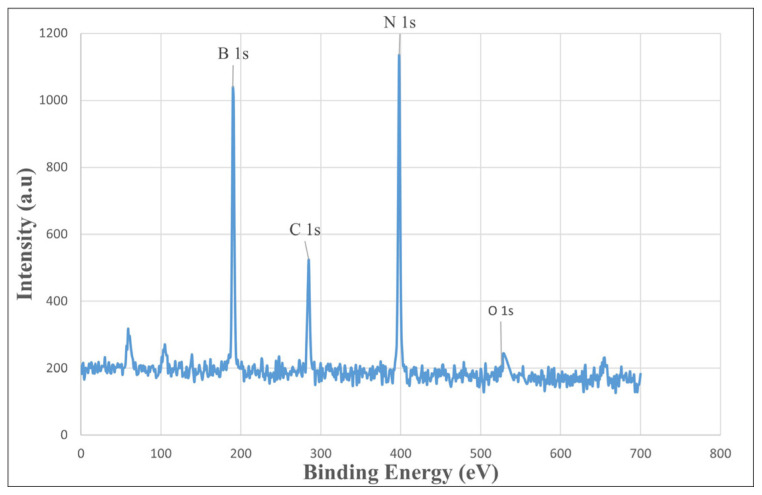
XPS spectrum of BNNTs. High-resolution spectra of B 1s, N 1s, C 1s, and O 1s are shown, indicating the elemental composition and chemical states of the functionalized nanotubes.

XPS further verified the elemental composition and purity of the synthesized BNNTs. The obtained spectra (Figure 11) clearly show the characteristic B 1s and N 1s peaks, confirming the formation of boron nitride without any detectable impurities and supporting the successful production of high-purity BNNTs [60]. The B 1s spectrum can be deconvoluted at a binding energy of 190.88 eV. This energy originates from the B–N bond and shows good agreement with the standard XPS value of BNNTs [60]. The N 1s spectrum corresponds to a binding energy of 397.81 eV and is attributed to the N–B bond. The C 1s peak was used as a calibration reference in the XPS analysis. The low-intensity O 1s peak is attributed to system-related or environmental sources.

**Figure 12:**
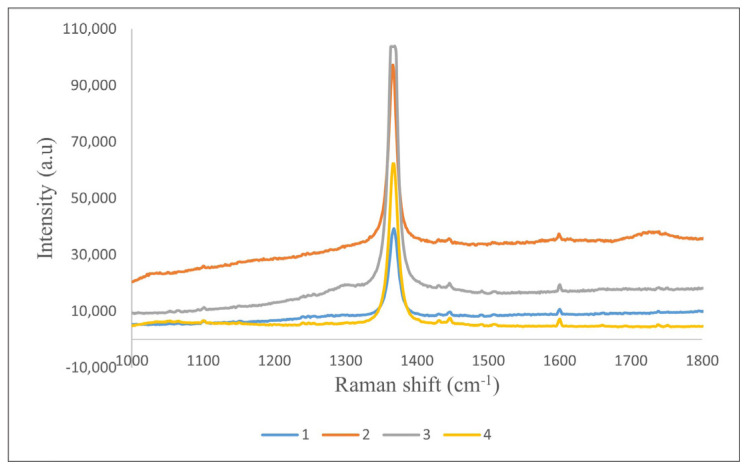
Repeatable Raman spectra of BNNTs. The vibration is observed at 1368 cm^−1^ corresponding to the E_2_g in-plane mode.

Raman spectra were collected from 4 randomly selected points to assess the structural uniformity of the BNNTs. As shown in Figure 12, the spectra were nearly identical across all points, confirming the directional and structural homogeneity of the product [41,56]. These comprehensive characterizations show that the synthesized BNNTs have superior properties, including high purity, excellent crystallinity and a remarkable aspect ratio of up to 7600. A sharp peak at 1368 cm^−1^ in the Raman spectrum indicates the E_2_g in-plane mode of h-BN and originates from the bond vibration between B and N on the same plane [41]. The E_2_g vibration peak can also be observed in the range of 1354–1366 cm^−1^ [41,59].

In this study, a reaction chamber model was proposed for the first time for the synthesis of BNNTs via the CVD technique (Figure 1 and Figure 2). This model was created by placing a curved quartz glass over a SiC template containing colemanite and a catalyst. The quartz glass allows NH_3_ gas to enter the reaction environment from the front, back and sides, thereby eliminating mass transfer limitations. This design enabled the synthesis of BNNTs from a more economical boron source, such as colemanite. In contrast, reaction chambers containing a quartz tube closed at one end are commonly used for BNNT synthesis via CVD [42,44,50,59]. In this study, a similar one-end-closed quartz tube was used for BNNT synthesis from colemanite; however, likely due to mass transfer limitations, BNNT formation did not occur. In a recent study, a BN crucible with Si/SiO_2_ templates positioned at angles of 60°, 90°, and 120° was used, successfully producing BNNTs [51]. The orientation of the templates at different angles affects the Fda, Frd, and Ftr forces. Trapping NH_3_ gas ensured its proper guidance during high-temperature BNNT synthesis from colemanite, facilitating effective BNNT formation. Our results indicate that the success of BNNT synthesis is directly related to the directional control of Fda, Frd, and Ftr forces, as previously mentioned [51]. The reaction chamber model, detailed here, is relatively simple and can be applied in large-scale BNNT production technologies. To date, no BNNT production method has been developed in this direction. This approach is potentially more efficient compared to the other BNNT production technologies discussed.

## 4. Conclusion

A surface-like growth strategy was effectively implemented to produce BNNTs with high density, aspect ratio, purity, and crystallinity. The synthesis using Fe_2_O_3_ and dual Fe_2_O_3_ + MgO catalyst systems was performed under a 300 sccm NH_3_ flow. The volatile BNNTs were confined within designated quartz-glass-covered regions on the SiC template inside a high-temperature furnace. The directional growth of BNNTs was successfully controlled by manipulating the vectorial force dynamics. As a result, subgram-scale quantities of pure BNNTs were synthesized from colemanite using Fe_2_O_3_ and MgO catalysts in a cost-efficient, single-step process. The BNNTs successfully synthesized through this method possess significant potential for future applications in various fields including polymer composite materials, biotechnology, and drug delivery systems as well as defense and aerospace industries.
